# Time‐restricted flight ability influences dispersal and colonization rates in a group of freshwater beetles

**DOI:** 10.1002/ece3.2680

**Published:** 2017-01-04

**Authors:** Lars Lønsmann Iversen, Riinu Rannap, Lars Briggs, Kaj Sand‐Jensen

**Affiliations:** ^1^ Freshwater Biological LaboratoryBiological InstituteUniversity of CopenhagenCopenhagen ØDenmark; ^2^ Amphi Consult ApSInternational Science Park OdenseOdense MDenmark; ^3^ Institute of Ecology and Earth SciencesUniversity of TartuTartuEstonia

**Keywords:** aquatic invertebrates, Coleoptera, colonization, oogenesis‐flight syndrome

## Abstract

Variation in the ability to fly or not is a key mechanism for differences in local species occurrences. It is increasingly acknowledged that physiological or behavioral mechanisms rather than morphological differences may drive flight abilities. However, our knowledge on the seasonal variability and stressors creating nonmorphological differences in flight abilities and how it scales to local and regional occurrences is very limited particularly for small, short‐lived species such as insects. Here, we examine how flight ability might vary across seasons and between two closely related genera of freshwater beetles with similar geographical ranges, life histories, and dispersal‐related morphology. By combining flight experiments of >1,100 specimens with colonization rates in a metacommunity of 54 ponds in northern and eastern Europe, we have analyzed the relationship between flight ability and spatio‐environmental distribution of the study genera. We find profound differences in flight ability between the two study genera across seasons. High flight ability for *Acilius* (97% of the tested individuals flew during the experiments) and low for *Graphoderus* (14%) corresponded to the different colonization rates of newly created ponds. Within a 5‐year period, 81 and 31% of the study ponds were colonized by *Acilius* and *Graphoderus*, respectively. While *Acilius* dispersed throughout the season, flight activity in *Graphoderus* was restricted to stressed situations immediately after the emergence of adults. Regional colonization ability of *Acilius* was independent of spatial connectivity and mass effect from propagule sources. In contrast, *Graphoderus* species were closely related to high connectivity between ponds in the landscape. Our data suggest that different dispersal potential can account for different local occurrences of *Acilius* and *Graphoderus*. In general, our findings provide some of the first insights into the understanding of seasonal restrictions in flight patterns of aquatic beetles and their consequences for species distributions.

## 1 Introduction

The ability of species to move between habitats is essential for their distribution (Gaston, 2009; Kokko & López‐Sepulcre, 2006). The spatial occurrence and temporal persistence of species are closely linked to their dispersal ability and dispersal strategy (Bowler & Benton, 2005; Clobert, Ims, & Rousset, 2004). Ultimately, these properties can shape the geographical ranges of species and determine continental diversity gradients (Baselga, Lobo, Svenning, Aragón, & Araújo, 2012; Geber, 2011; Svenning & Skov, 2004). Thus, understanding how dispersal evolves and persists among and within species in a changing environment is a key element when evaluating both current and future drivers of biodiversity change (Pereira et al., 2010; Urban, 2015).

Although many processes such as environmental stability, kin competition, and inbreeding influence dispersal traits (Matthysen, 2012; Ronce, 2007; Starrfelt & Kokko, 2012), abiotic barriers are often considered to be the main evolutionary drivers of dispersal (Baguette, Blanchet, Legrand, Stevens, & Turlure, 2013; Kubisch, Holt, Poethke, & Fronhofer, 2014). Usually, environmental disturbance increases dispersal provided that suitable habitats are evenly distributed (Gadgil, 1971; Levin, Cohen, & Hastings, 1984; Poethke, Hovestadt, & Mitesser, 2003). However, high dispersal rates come at a cost. When habitat quality varies spatially, a high level of dispersal can lead to emigration from optimal habitats and may reduce the use of high‐quality habitats (Bonte et al., 2012; Holt, 1985; North, Cornell, & Ovaskainen, 2011). In theory, this mechanism is further enhanced when the mortality risk during dispersal is high, for example, when island species have to cross open waters or aquatic species move across hostile terrain (Bonte et al., 2012; Kubisch et al., 2014). Spatial isolation and local habitat conditions have been shown to alter intraspecific dispersal properties either as a consequence of local habitat connectivity (Hanski, Erälahti, Kankare, Ovaskainen, & Sirén, 2004; Soons & Heil, 2002) or at the front edge of expanding range margins (Simmons & Thomas, 2004; Thomas et al., 2001).

Traditionally, differences in dispersal properties within and between species have been studied by analysis of different morphological structures related to the dispersal organs of species such as wing structure or flight musculature (Hargreaves & Eckert, 2014). But morphological dispersal traits do not necessarily explain species occurrences (e.g., Grönroos et al., 2013; Schulz, Siqueira, Stefan, & Roque, 2012), and dispersal events driven by physiological or behavioral factors with no relationship to morphological traits are well documented among larger vertebrates (Bekoff, 1977; Duckworth & Badyaev, 2007). These physiological or behavioral factors have also been proposed to be important for invertebrate species (Clobert, Galliard, Cote, Meylan, & Massot, 2009; Hanski et al., 2004), yet we know very little about the seasonal variability and stressors generating nonmorphological differences in flight abilities and how this may influence local and regional occurrences of invertebrate species (Bilton, 2014; Bilton, Freeland, & Okamura, 2001).

In this study, we aimed to demonstrate how nonmorphological differences in flight abilities might vary across season and how these differences scale to colonization rates of newly created habitats in five invertebrate species. We examined this using a group of aquatic beetles distributed within distinctively isolated habitats (lakes and ponds). As a consequence of the spatial isolation and short geological lifetime of freshwaters, aquatic beetles have undergone an evolutionary selection toward strong dispersal abilities during the invasion of these habitats (Arribas et al., 2012; Bilton et al., 2001). Following the invasion, subsequent reduction in dispersal has been reported for several species (Jackson, 1952, 1956), offering an opportunity for studying differences in flight abilities both within and between species. Using experiments and field observations, we studied five species of European diving beetles from two genera, *Acilius* and *Graphoderus* (Coleoptera: Dytiscidae), with similar geographical ranges, food resources, and life histories (Bergsten & Miller, 2005; Nilsson & Holmen, 1995), but contrasting regional occurrences in Europe (Foster, 1996; Foster & Bilton, 2014). Both genera have well‐developed wings and flight musculature (Bergsten & Miller, 2005; Kehl & Dettner, 2007), but contrasting flight capabilities (Foster, 1996; Kehl & Dettner, 2007). All five study species are associated with richly vegetated standing waters (Miller & Bergsten, 2016; Nilsson & Holmen, 1995), and the two genera often co‐occur together in the same lakes and ponds (Nilsson, Elmberg, & Sjoberg, 1994). Within the study region of interest, the three *Graphoderus* species are most often found in larger permanent habitats, contra the two *Acilius* species which are omnipresent occupying both large and small habitats (Iversen, Rannap, Thomsen, Kielgast, & Sand‐Jensen, 2013; Lundkvist, Landin, & Milberg, 2001).

Within this framework, we firstly tested whether differences in flight ability are species specific or clustered within the two genera. During three time periods, covering species' life cycles, we experimentally tested individual flight ability of each species. If differences in dispersal have been the evolutionary mechanism behind the split between *Acilius* and *Graphoderus*, we would expect the seasonal patterns in flight abilities to be conditional on the two genera studied and not species specific. Secondly, we examined the hypothesis that differences in dispersal potential scales to local occurrences by detecting the colonization rates of our study species within 54 newly created or restored ponds. If difference in flight ability does affect colonization probability, we would expect concordance between the observed experimental flight abilities and in situ colonization rates.

## 2 Methods

### 2.1 Flight experiments

In order to quantify the potential ability to fly within the five study species, we performed ex situ flight experiments. The setup consisted of 20–25 four‐liter containers with only one possible exit point, either flying from a vertical or a horizontal position (Fig. S1). The experiment measured the ability to fly or not to fly, and it did not record flight distance per se. Adult diving beetles were collected, and the experiments were conducted in late spring (end of May 2011 and 2015), mid‐summer (first half of July 2015), and early autumn (first half of September 2011 and 2015). The sampling periods represented three different phases of the species' life history: (1) the period just after emergence from the pupae (summer), (2) the prehibernation period when beetle activity is related to foraging (autumn), and (3) the breeding period (spring). The containers were placed in transparent plastic boxes which were moved between a glasshouse and an outside area in order to keep the ambient daytime temperatures between ~20 and 35°C, and nocturnal temperatures above 10°C. The temperature range covers temperatures promoting dispersal activity in aquatic beetles (Csabai, Kálmán, Szivák, & Boda, 2012). Individuals were collected at 16 different sites in southern Sweden and eastern Denmark (Table S1). During the 2 years of study, a total of 1,128 individuals were collected and tested (Table S1). Following field sampling, all animals were acclimatized for 2–3 days prior to the experiments. High‐quality tap water derived from groundwater reservoirs was used during the acclimatization and experiments to exclude any potential fledge caused by toxins or chemical traces from predators. The experiments were conducted for 14 days with a constant density of 10 specimens per container. The trials were conducted separately for each species and sex. The animals were not fed before or during the experiment, and any dead or dying animals were removed immediately from the experiments and excluded from further analysis. All collected specimens of the strictly protected *G. bilineatus* were released to their respective localities after the experiment. Specimens of the other common species were killed in 96% ethanol and checked for the presence of well‐developed wings and flight musculature. All of the specimens used in the experiments had well‐developed wings and flight musculature.

In order to confirm that our setup was appropriate to determine differences in flight ability between different species of diving beetles, we performed flight experiments on 13 additional species (Table S2).

### 2.2 Colonization of newly created and restored ponds

The ability to colonize new habitats was tested within a network of 3‐ to 5‐year‐old restored or newly created ponds in the Haanja landscape park, southern Estonia. In 2006, new ponds were created and completely overgrown ponds reactivated through conservation actions, for example, by removing bushes, tall and dense vegetation, and muddy sediment (Rannap, Lõhmus, & Briggs, 2009). The land use in the area is a mixture of open, extensively used farm‐ and grassland and mixed forest. All five study species are common in the natural lakes, cattle ponds, and beaver floods scattered throughout the landscape (L. L. Iversen personal observations). In mid‐June 2011, we recorded the presence of the two study genera in 54 restored or newly created ponds. Due to the late sampling date, adult beetle abundance was expected to be low and the presence of larvae was used as the measure of colonization. Identification of larvae was restricted to genus, because intraspecific morphological characters within both *Acilius* and *Graphoderus* have not yet been described (Mogens Holmen personal communication). A semistandardized dipnetting method was used to detect the presence or absence of the study organisms (Iversen et al., 2013). Diving beetle larvae were actively searched for during a 45‐min period at each site by sweeping a hand dipnet (40 × 40 cm frame) through the vegetation and detrital material. Along with larvae occurrence, we recorded vegetation cover, structured in four density classes, and shading from surrounding trees (see Appendix S1 for details).

### 2.3 Analysis of data

Applying linear contrast models to our data, differences in flight ability between *Acilius* and *Graphoderus* were evaluated. When tested explicitly, *p*‐values related to the effect of explanatory variables were evaluated at a 5% significance level and, unless stated otherwise, correspond to a chi‐squared test. Reported confidence intervals correspond to 95% likelihood confidence intervals.

Using the observed ability to leave the experimental setup by flight as the response variable (coded as 1, flying, or 0, nonflying), differences in flight ability were tested across individuals with a linear logistic regression model via a logit‐link function. Genus (*Graphoderus* or *Acilius*), season (spring, summer, or autumn), and the interaction between these factors were defined as the explanatory variables. Initial models including species, sampling year, and site as explanatory variables produced the same significance of genera and season as the models reported here. Nor were there any differences in flight ability between sexes (same model but with sex of the individual as the only explanatory variable, *p* = .50). Within genera, differences in flight ability between species were evaluated in a linear logistic regression model. The time spent in the experimental setup before flying away was modeled by a Gaussian linear mixed model and tested by a likelihood ratio test; genus was used as a fixed factor, species and sites as random factors.

Differences in colonization ability between *Acilius* and *Graphoderus* in the newly created and restored ponds (presence/nonpresence at each site) were evaluated by a mixed linear logistic regression model via a logit‐link function. Genus and restoration measure (restored or newly created) were used as fixed factors, while site was used as a random factor. Using an outlying mean index analysis (OMI) (Dolédec, Chessel, & Gimaret‐Carpentier, 2000), we tested whether the observed colonization patterns were independent of the habitat characteristics (within the studied localities). OMI analysis provided a measure of niche position and niche breadth from a subset of localities, compared to the environmental space present in a region. A principal component analysis (PCA) was performed on the *z*‐scores of the four vegetation variables and the level of shading at each site. This PCA was used as the sampling unit to calculate the OMI and related to the occurrence matrix of the two genera. The observed OMI value was compared to the random OMI value generated from randomly selected sites with the same frequency of occurrence as *Acilius* and *Graphoderus*. Using a Monte Carlo test generated from 1000 null‐model repetitions, *p*‐values were tested whether or not the observed OMI value was greater (more niche specific) than expected by chance. We created a local proximity index (Gustafson & Parker, 1994) for each sampling pond based on the size of and euclidian distance to all other lakes and ponds within 5 km of the given pond. The index incorporates both isolation and the size of potential propagules [mass effect (Leibold et al., 2004)] by dividing area with the squared distances to the sampling pond and summing this for all lakes and ponds within the search radius of each sampling pond (Whitcomb et al., 1981). We tested for differences in relationship between colonization probability and site proximity index between *Acilius* and *Graphoderus* using linear logistic regression model via a logit‐link function, including the interaction and fixed term of the proximity index and genera as explanatory variables and the observed presence/nonpresence as response variable.

All analyses were conducted in R ver. 3.2.0 using the additional packages nlme, lme4, and ade4.

## 3 Results

### 3.1 Flight experiments

**Figure 1:**
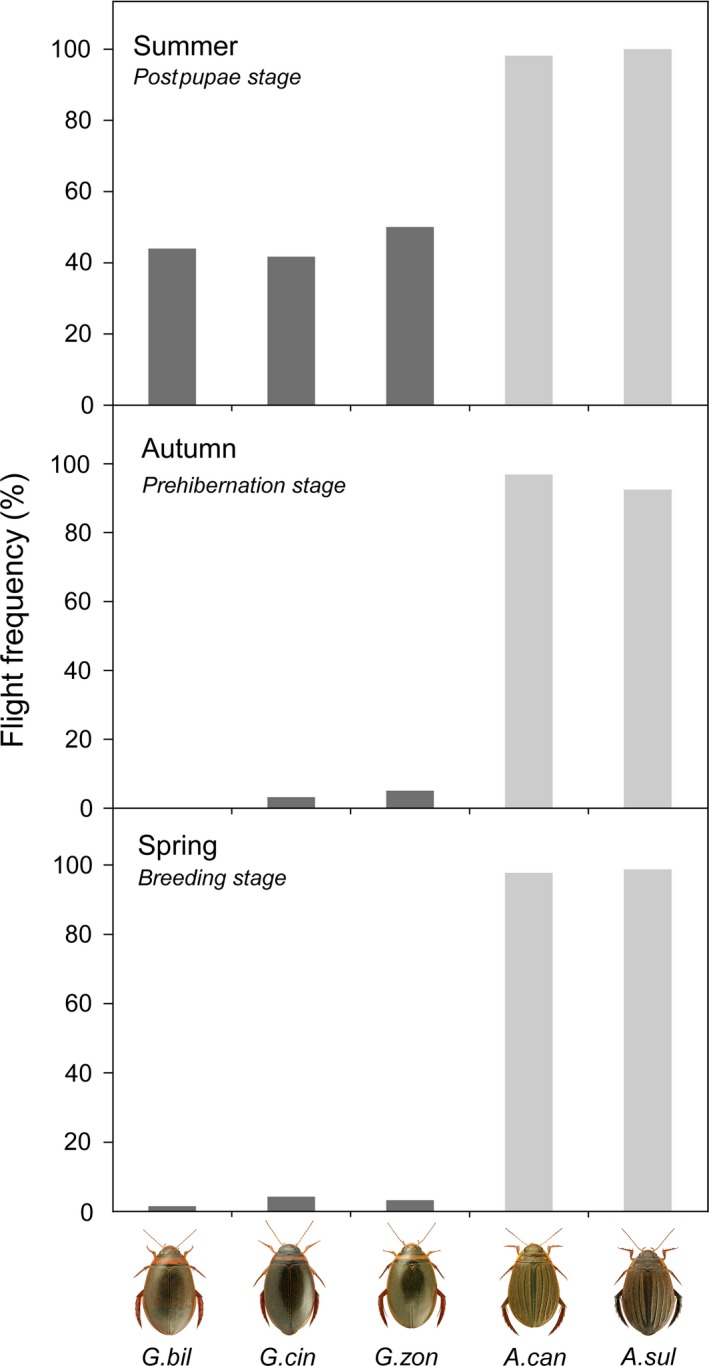
Dispersal properties of *Acilius* (gray) and *Graphoderus* (black). The percentage of individuals flying during the experimental trials. The experiments were conducted across three different seasons: just after postpupae state in July (summer), prior to hibernation in September (autumn), and posthibernation during the main reproductive period in May (spring). A total of 1,128 individuals were examined: *A. canaliculatus n* = 242, *A. sulcatus n* = 164, *G. bilineatus n* = 232, *G. cinereus n* = 338, and *G. zonatus n* = 152

**Figure 2:**
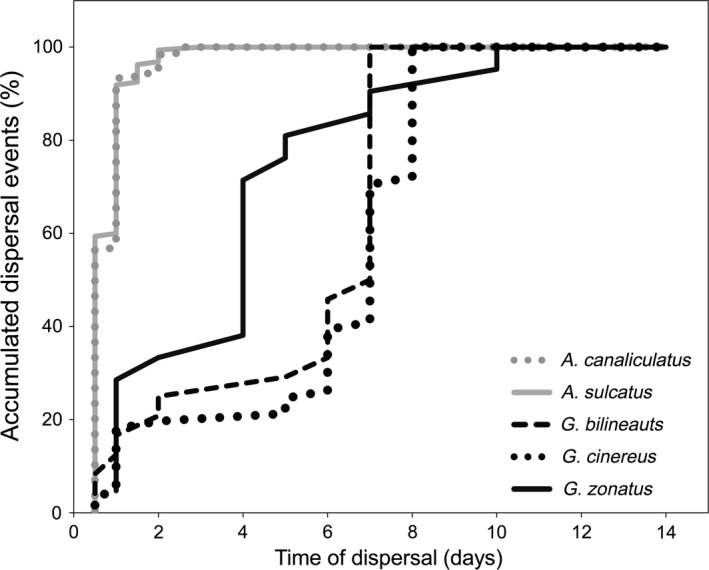
Accumulated dispersal events after experimental introduction across the three study periods of *Acilius* (gray) and *Graphoderus* (black). The accumulated percentages refer to the number of flying individuals per species. *A. canaliculatus n* = 239, *A. sulcatus n* = 159, *G. bilineatus n* = 24, *G. cinereus n* = 61, and *G. zonatus n* = 21

Flight experiments showed profound differences in flight ability between the two genera. Across seasons, flight ability was high for *Acilius* (97% of the tested individuals flew during the experiments) and low for *Graphoderus* (14%; Figure 1) and there was a significant interaction between genera and season (|χ^2^| = 8.3, *df* (degrees of freedom) = 1121, *p* < .05). Between the two *Acilius* species, there was no difference in flight ability across species ((|χ^2^|=1.1, *df* = 400, *p* = .52), across seasons (*p* = |χ^2^| = 2.1, *df* = 402, *p* = .36), or for that matter between seasons across species (|χ^2^|=2.3, *df* = 402, *p* = .32). In contrast, the three *Graphoderus* species showed a systematic change between the seasons for all species (|χ^2^|=176.3, *df* = 716, *p* < .001), but no difference between species (|χ^2^|=1.5, *df* = 716, *p* = .47). *Graphoderus* species had a distinct peak in flight in summer (dispersal rate 45%) and almost no flight for the rest of the year (dispersal rate 3%) (Figure 2, Table S1).

The time spent in the experimental setup differed significantly between the genera (|LRT| (likelihood ratio test) = 376.6, *df* = 499, *p* < .001). *Acilius* flew almost immediately (within 0.78 [0.74; 0.82] days; mean and 95% CL) after being introduced, while on average, the dispersing individuals of *Graphoderus* left the containers after 5.37 [4.86; 5.88] days (Figure 2).

The setup documented flight events across a large range of additional species without detecting any systematic restrictions (Table S2). Thus, we are confident that the results reflect true differences in flight ability between the two genera and similarities of species within genera.

### 3.2 Colonization of newly created and restored ponds

**Figure 3:**
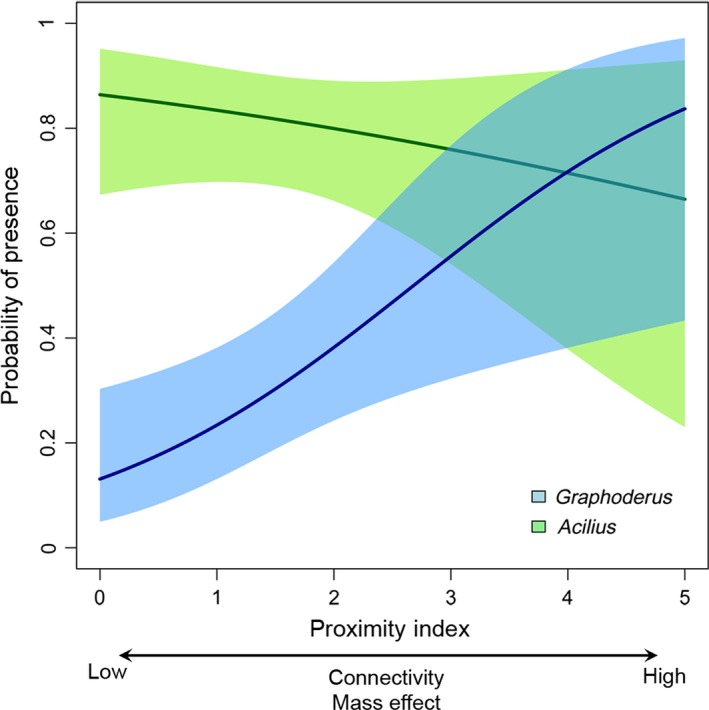
Probability of presence in 54 newly created Estonian ponds of *Graphoderus* (blue) and *Acilius* (green) in relation to landscape proximity index. The central tendency is shown by the solid line, and 95% confidence limits are shaded

Individuals of *Acilius* and *Graphoderus* had colonized 81% and 31% of the 54 “new” Estonian ponds, respectively. *Acilius* showed a significantly higher likelihood of colonizing the sites than *Graphoderus* (|χ^2^|= 28.9, *df* = 106, *p* < .001). The OMI estimation for the two genera showed no difference other than expected by random distribution (*Acilius p* = .14 and *Graphoderus p* = .74). Hence, habitat availability and possible differences in habitat conditions between sites did not influence colonization rates. There was a significant difference in probability of occurrence along a connectivity gradient between the two genera (|χ^2^|= 5.9, *df* = 104, *p* < .05). *Graphoderus* showed a higher likelihood of occurrence with an increase in the proximity index (relative change in odds by a factor of 2.56 [1.21; 5.55], Figure 3), whereas *Acilius* occurrence was independent of landscape structure (odds changed by a factor of 0.79 [0.47; 1.40], Figure 3).

## 4 Discussion

Our results document that after being placed in the experimental setup, the two *Acilius* species flew immediately at all times, while the three *Graphoderus* species only dispersed in the postpupae state. High dispersal rates of *Acilius* throughout the season resemble the dispersal patterns of other highly dispersive diving beetles (Boda & Csabai, 2013; Lundkvist, Landin, & Karlsson, 2002; Miguélez & Valladares, 2008). In contrast, the restricted dispersal events in *Graphoderus* may be related to the oogenesis‐flight syndrome (Dingle, 1972). Individuals disperse in the premature state, then settle, and allocate energy for reproduction and thereby create the observed single peak in dispersal activity (Fronhofer, Poethke, & Dieckmann, 2015). So far, very few studies have documented the oogenesis‐flight syndrome in aquatic beetles (Landin, 1980), and our results provide insights into the understanding of seasonal restrictions in flight patterns of aquatic beetles (Bilton, 2014).

Both the flight experiments and the colonization rates supported the expectations of a mobility syndrome, where a high dispersal potential leads to higher dispersal rates within populations (Cote, Clobert, Brodin, Fogarty, & Sih, 2010; Ducatez et al., 2012). When dispersing, in any of the three study periods, *Acilius* showed a markedly higher dispersal rate than *Graphoderus* and also a higher colonization rate of the newly created Estonian ponds. During summer, only 45% of the *Graphoderus* specimens left the experimental containers. This could be interpreted as a conservative dispersal strategy according to which populations are polymorphic and include both flying and nonflying individuals (Landin, 1980; Vepsäläinen, 1978). Given the short time span during which dispersal is possible and the high mortality risk during emigration, having a proportion of nonflying individuals might increase the likelihood of population survival.

The permanent, well‐developed wings and flight muscles of both *Acilius* and *Graphoderus* suggest that the observed differences in the ability to fly were not driven by morphological differences. The slow dispersal of all three *Graphoderus* species in the experiments suggests that dispersal decisions are a conservative choice within this genus. Other studies have highlighted that variation in flight abilities could be a consequence of differences in physiological tolerances, resources utilizations, and local competition (Baguette, Clobert, & Schtickzelle, 2011; Hanski et al., 2004; King & Roff, 2010). Future work should aim to clarify the underlying triggers leading to the different flight abilities between *Acilius* and *Graphoderus*.

The correlation between colonization probability of *Graphoderus* and the proximity index in the Estonian ponds suggests that both mass effect and connectivity promote the occurrence of the genera (Heino et al., 2015; Iversen et al., 2013). In contrast, the chance of *Acilius* colonizing any of the study ponds was independent of the property of the surrounding landscape (at least within the spatial scale of this study). This result could suggest that not only are there differences in dispersal rates between the two species but also in potential dispersal distances during flight. However, specific information on dispersal distances from propagule sources would be needed in order to differentiate the landscape‐dependent effect of potential dispersal distance contra the frequency of flight events in *Acilius* and *Graphoderus*.

Our results can also explain the regional conservation status of the studied species. All three *Graphoderus* species are threatened locally in many regions and have even become extinct in some countries, while the two *Acilius* species are common throughout their range (e.g., Bergsten & Miller, 2005; Cuppen, Koese, & Sierdsema, 2006; Foster, Bilton, & Nelson, 2016). It is likely that this rarity of the *Graphoderus* species is a consequence of the high turnover rate and destruction of freshwater habitats caused by anthropogenic activities in western Europe (Foster, 1996; Foster & Bilton, 2014). The restricted dispersal of the *Graphoderus* species could limit their ability to track these habitat changes, leading to the decline in population numbers and extinction. In contrast, the two *Acilius* species might be able to track these environmental changes, partly due to a strong dispersal ability. Our results support previous findings highlighting the importance of integrating landscape connectivity and stability into conservation strategies of species such as the three studied *Graphoderus* species (Iversen et al., 2013) and suggest that such approaches are important even for conservation on a local scale.

In summary, our study shows distinct differences and seasonal constraints in flight ability within a group of diving beetles. Given the clear contrast between genera and the remarkably similar dispersal properties among species of the same genera, our data suggest that species flight ability is important for profound differences in local occurrences between *Acilius* and *Graphoderus*. The two *Acilius* species dispersed continuously throughout the season and where independent of spatial connectivity and mass effect. In contrast, the short window during which dispersal took place in all three *Graphoderus* species scaled to differences in dispersal rates, within and across seasons, and differences in colonization rates between the two genera.

Our findings provide some of the first insights into the understanding of seasonal restrictions in flight patterns of aquatic beetles and invertebrates in general.

## Conflict of interest

None declared.
