# Supplementary material for: Time‐restricted flight ability influences dispersal and colonization rates in a group of freshwater beetles
Source: Ecol Evol. 2017 Jan 4;7(3):824–30. doi: 10.1002/ece3.2680 (PMC5288253; doi:10.1002/ece3.2680)
Supplement: Supplementary file 1 [file ECE3-7-824-s001.docx]

Supplementary material:

**Appendix S1:**

**Habitat and spatial variables used in the study:**

Habitat descriptions were conducted along with the collection of species data. Instead of recording fluctuating water-chemistry variables, we focused on more stable physical and biological variables. Variables assumed to influence presence/absence of the different Aciliini species were selected based on existing literature and results of preliminary fieldwork conducted on the group.

At each site, shading was defined as the percentage of the shoreline having standing trees of more than two meter in height, present within three meters from the shoreline. The shallow areas (distance from the banks with a depth < 30 cm) were estimated as the maximum extent for each of the four cardinal banks. Following Iversen et al. (2013) larval and adult habitats were obtained by visually estimating the percentage vegetation cover of the total lake surface area. Vegetation structure were subdivided into 4 classes representing submerged vegetation, floating vegetation, riparian vegetation < 1 meter in height, and riparian vegetation > 1 meter in height. Surface area of ponds (< 0.5 ha ) was measured in the field, while lakes (> 0.5 ha ) were measured prior to the fieldwork, using high resolution orthophotos. All ponds and lakes in the study region was geolocated via high resolution orthophotos.

References

Iversen, L. L., Rannap, R., Thomsen, P. F., Kielgast, J., & Sand‐Jensen, K. (2013). How do low dispersal species establish large range sizes? The case of the water beetle *Graphoderus bilineatus*. Ecography, 36(7), 770-777.


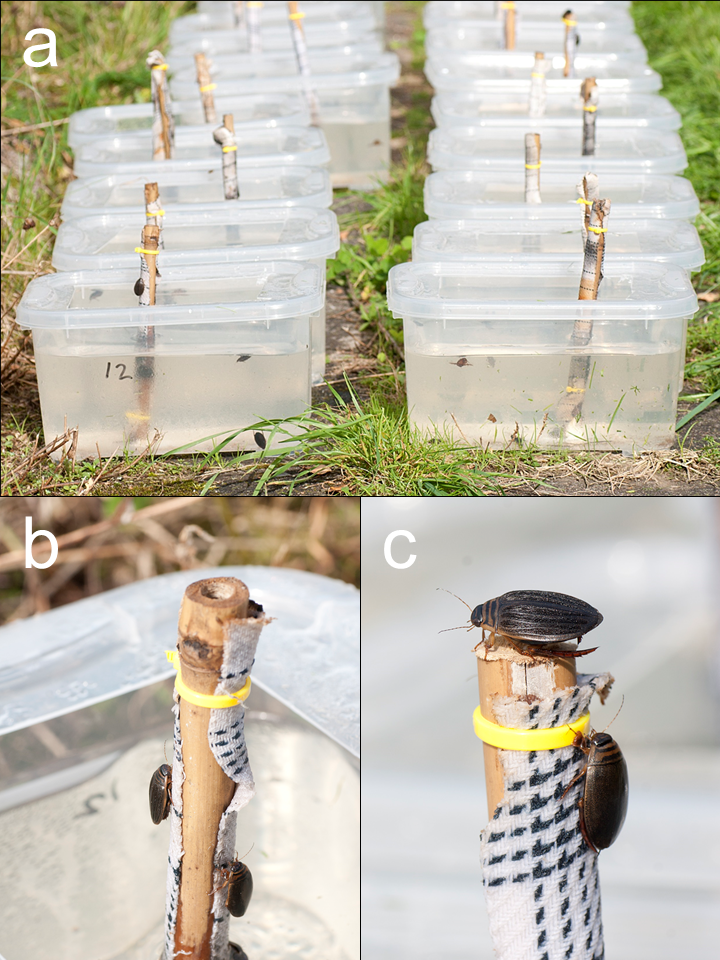


Fig S1: Ex-situ flight experiments of the study species were conducted in 4 -litre containers (a) with only one possible exit point (b), either flying from a vertical or a horizontal position (c).

.

*Table s1***:** Study sites and number of animals tested in the dispersal experiments. The number in brackets refers to the amount of animals flying during the experiment

*Table S2***:** Results from flight experiments conducted on 13 other species of diving beetles. All trials were performed during the spring of 2011.

| **Species** | **Nr. of individuals tested** | **Nr. of observed flights** |
| --- | --- | --- |
| Colymbetes paykulli | 18 | 18 |
| Dytiscus lapponicus | 14 | 0 |
| Dytiscus marginalis | 6 | 5 |
| Hydaticus aruspex | 6 | 5 |
| Hydaticus seminiger | 12 | 10 |
| Hydaticus transversalis | 2 | 1 |
| Hyphydrus ovatus | 4 | 1 |
| Ilybius fuliginiosus | 3 | 0 |
| Rhantus exoletus | 3 | 3 |
| Rhantus frontalis | 1 | 1 |
| Rhantus grapii | 8 | 6 |
| Rhantus notaticollis | 6 | 6 |
| Rhantus suturalis | 3 | 2 |
